# Development of Forensically Important *Megaselia scalaris* and *Dohrniphora cornuta* (Diptera: Phoridae) in Sandy Loam Under Constant Moisture and Different Temperature Regimes

**DOI:** 10.3390/insects16080760

**Published:** 2025-07-24

**Authors:** Wei Han, Dianxing Feng, Yanan Tang

**Affiliations:** College of Life Science and Engineering, Shenyang University, Shenyang 110044, China; hw06122024@163.com (W.H.); tyn0986@163.com (Y.T.)

**Keywords:** phorid fly, post-burial interval, development time, body length, intra-puparial period

## Abstract

The Phoridae are relatively common insects on buried corpses, and their developmental information plays an important role in estimating post-burial interval (PBI) for buried corpses. Developmental time, larval body length, and intra-puparial developmental changes in two forensically important phorid flies, *Megaselia scalaris* (Loew, 1866) and *Dohrniphora cornuta* (Bigot, 1857) (Diptera: Phoridae), were studied in sandy loam with 20% moisture content at 18, 21, 24, and 27 °C. The juvenile development time of both species decreased with increasing temperature. At 18, 21, 24, and 27 °C, the larval development time of *M. scalaris* was 165.18 ± 2.96 h, 119.72 ± 2.74 h, 90.28 ± 3.54 h, and 63.04 ± 3.45 h, respectively, with corresponding intra-puparial development times of 606.67 ± 3.38 h, 404.62 ± 3.28 h, 269.22 ± 6.04 h, and 237.57 ± 3.41 h. For *D. cornuta*, the larval development time was 249.37 ± 4.88 h, 154.59 ± 1.81 h, 108.80 ± 2.65 h, and 86.04 ± 3.91 h, respectively, while the intra-puparial development time was 593.37 ± 4.75 h, 414.23 ± 3.51 h, 261.02 ± 3.16 h, and 236.52 ± 2.66 h. The larval body length of both species showed a cubic curve relationship with development time. Based on the morphological characteristics, the intra-puparial period of both species were divided into 12 substages. As the temperature increased, the time required for both species to reach each substage decreased significantly. The present study enriches the developmental information of the two Phoridae species and provides a reference basis for using these two phorid flies to estimate the post-burial interval (PBI) of corpses in China.

## Simple Summary

Burial is a common way to dispose of corpses. Because of the soil barrier, smaller phorid flies show greater capability to reach buried corpses compared to larger fly species, thereby becoming the predominant insects associated with buried remains. Data on insect development under burial conditions are currently lacking. This study established the developmental data (duration, larval body length, and intra-puparial developmental variation) for two important forensic insects, *Megaselia scalaris* (Loew, 1866) and *Dohrniphora cornuta* (Bigot, 1857) (Diptera: Phoridae), which were kept in sandy loam with a moisture content of 20% at various constant temperatures. Both species’ development in the soil was accelerated by rising temperatures. The two phorid flies’ development data obtained in this study provided a reference for the estimation of post-burial interval (PBI) of corpses.

## 1. Introduction

The Phoridae, commonly known as scuttle flies, comprises more than 4300 described species in the world [1]. They are usually small individuals, mostly ranging from 1.5 to 3.0 mm in length, and may feed on a variety of decaying organic matter [2,3,4]. Some species in this family have been found associated with vertebrate carrion and are therefore considered forensically important [5]. To date, larvae and pupae of 23 species in 10 genera of the family of Phoridae have been collected on human and animal remains, as well as animal muscle tissues [6,7,8,9,10,11,12,13,14,15,16,17,18,19,20,21,22,23,24,25,26,27] (Table 1).

The use of developmental data of necrophagous insects collected from human corpses at crime scenes to estimate the time of death is a common method used in forensic entomology [28]. Currently, developmental data of *Megaselia scalaris* (Loew, 1866) [10,29,30,31], *M. abdita* Schmitz, 1959 [10], *M. spiracularis* Schmitz, 1938 [32,33,34], *Dohrniphora cornuta* (Bigot, 1857) [4,35], and *Diplonevra funebris* (Meigen, 1830) [36] under different constant temperature and humidity conditions have been established, while *D. peregrina* (Wiedemann, 1830) [37], *M. rufipes* (Meigen, 1804), and *M. giraudii* (Egger, 1862) [26] constructed developmental data under naturally fluctuating temperatures. However, developmental data in soils are lacking for several phorid flies frequently found on buried corpses, such as *Conicera tibialis* Schmitz, 1925 [7], *C. similis* Haliday, 1833 [8], *M. abdita* [18], *M. scalaris* [14,15,16], and *D. cornuta* [9].

The soil type and moisture are two important environmental parameters influencing the development, survival, and spatial distribution of soil-dwelling insects [38,39,40,41,42]. In forensic entomology, Kökdener and Şahin Yurtgan [43] reported that soil type (clay, loamy, and sandy) and moisture content (0, 25, 50, 75, 100%) had a significant effect on the development time and larval and pupal survival of *Lucilia sericata* (Meigen, 1826) in soil at 27 °C. Similarly, the results of Han et al. [44] indicated that soil types, soil moisture, and their interaction significantly influenced the growth and development of *M. scalaris* and *D. cornuta*, with moisture being the most influential factor. In each soil, 20% and 40% moisture content were more suitable for their development. Developmental duration and maximum larval body length varied significantly depending on soil type and moisture content. However, their results were obtained at a constant temperature (27 °C) and lack data on intra-puparial development, which is insufficient for practical post-burial interval (PBI) estimation [43,44].

*Megaselia scalaris* and *D. cornuta* are the common phorid flies found on indoor corpses [6,9,10,11,12,13], and they can also be the main insects on buried corpses [9,14,15,16]. When using their developmental data to estimate the PBI of buried corpses in forensic investigations, it is crucial to consider the effects of soil type and moisture content to avoid inaccurate estimations. Therefore, in this study, we established data on larval and intra-puparial development time, larval body length, and intra-puparial developmental changes in *M. scalaris* and *D. cornuta* in sandy loam with 20% moisture content at temperatures of 18, 21, 24, and 27 °C, aiming to provide a reference for using these two phorid flies to estimate the PBI of buried corpses.

## 2. Materials and Methods

### 2.1. Insects

*Megaselia scalaris* and *D. cornuta* used in this study were from the stock colonies that had been kept for over 10 generations in the laboratory. The two laboratory colonies, established from adult specimens baited with lean pork in the canteens at Shenyang University (123°27′39″ E, 41°49′27″ N), Shenyang City, Liaoning Province, China, were maintained in an artificial climate incubator (Ningbo Laifu Technology Co., Ltd., Ningbo, China) with a temperature of 21–24 °C, 75% relative humidity, and 12 L:12 D photoperiod. To mitigate genetic diversity loss due to long-term maintenance, new individuals collected from the same location were introduced into rearing phorid flies.

### 2.2. Soil

Sandy loam (sand: 49.01%; silt: 48.24%; clay: 2.75%; organic matter: 1.77%; pH 6.01) was used in this study. The soil was collected from Shenyang City, Liaoning Province, China (123°26′49″ E, 41°54′39″ N). The soil moisture content was calculated using the formula provided by Chen and Shelton [45]:

Soil moisture (%) = [weight of distilled water added/(weight of saturated soil − weight of dry soil)] × 100%

### 2.3. Observation on Development of Necrophagous Phorid Flies

Five to ten pairs of adult flies of both species were reared in 1000 mL narrow-necked bottles (Sichuan Shubo Co., Ltd., Chongzhou, China) sealed with industrial filter cloth (Suzhou Tebang Environmental Protection Technology Co., Ltd., Suzhou, China), with fresh lean pork provided as a food source in artificial climate incubators (Ning-bo Laifu Technology Co., Ltd., Ningbo, China) set to 18 °C, 21 °C, 24 °C, or 27 °C, 75% relative humidity, and 12 L:12 D photoperiod. Eggs were collected from the bottles and placed in Petri dishes, which were then transferred to the incubator for periodic hatching observation. A total of 1600 first instar larvae from each species were cautiously removed using a soft brush after the eggs hatched and placed on fresh lean pork. Collections lasted for half an hour. Therefore, time zero was egg hatching + 30 min maximum. After post-feeding larval pupariation, the prepupae were picked from the bottles. Collections lasted for 1 h. Time zero was consequently prepupa formation + 1 h maximum. First instar larvae (with lean pork) or prepupae were subsequently transferred to clear plastic bowls (upper diameter: 11.8 cm, base diameter: 6.8 cm, height: 6.5 cm) containing soil with 20% moisture content. To prevent water evaporation, a plastic lid was placed over each bowl. The bowls were then placed back into the previous artificial climate incubator. Developmental progress was observed regularly with 10 replicates per test condition. Developmental stages were defined as follows:

(1) Larval feeding period: from egg hatching to first larval departure from pork tissue.
(2) Larval period: from egg hatching to first larva pupariation.
(3) Intra-puparial period: from prepupa formation to first adult emergence.

### 2.4. Measurement of Larval Body Length

Ten larvae of both species were randomly sampled at regular intervals from hatching until the first larva initiated pupariation at 18, 21, 24, and 27 °C. For *M. scalaris*, the larvae were randomly sampled every 6 h. To ensure sufficient specimens, particularly under lower temperatures, the sampling interval for *D. cornuta* was set at 12 h. The collected larvae were killed with hot water and preserved in 75% alcohol. The larvae were placed under an Olympus BX41 stereomicroscope (Tokyo, Japan). Pictures were taken using an Olympus DP-71 digital camera (Tokyo, Japan) and DP Controller 3.1 software. The larval body lengths in each picture were measured using the measurement tool in Image-ProPlus 6.0 software.

### 2.5. Morphological Changes During the Intra-Puparial Period of Both Species

We followed the experimental method of Feng and Liu [30]. The pupae were reared at 18, 21, 24, and 27 °C. Ten pupae were removed from the soil for observation at 2 h intervals (24 °C and 27 °C) or 4 h intervals (18 °C and 21 °C) throughout their metamorphosis. The process was repeated three times at each temperature. The pupae were submerged in 1% sodium alginate solution in a Petri dish. The puparium was removed from each pupa using insect needles under an Olympus BX41 stereomicroscope. Pictures were taken using an Olympus DP-71 digital camera and DP Controller 3.1 software. All the above operations were completed within 30 min.

### 2.6. Statistical Analysis

Data analysis was performed using SPSS 27.0 and Graphpad Prism 9.5 software. The development time of larval and intra-puparial periods, along with larval body length between different temperatures in sandy loam with 20% moisture content, were compared using one-way ANOVA, followed by Tukey’s HSD (honestly significant difference) post hoc test. Statistical significance was defined as α = 0.05 for all analyses. The relationships between larval body length and post-hatching development time were assessed through regression analyses.

## 3. Results

### 3.1. Development Time of Larvae and Pupae of Two Necrophagous Phorid Flies

The larval and intra-puparial development time, pupation rate, and emergence rate of the two phorid flies in sandy loam with 20% moisture content at 18 °C, 21 °C, 24 °C, and 27 °C were shown in Table 2 and Table 3. The shortest larval development time of *M. scalaris* was 165.18 ± 2.96 h, 119.72 ± 2.74 h, 90.28 ± 3.54 h, and 63.04 ± 3.45 h, respectively, while the shortest intra-puparial development time was 606.67 ± 3.38 h, 404.62 ± 3.28 h, 269.22 ± 6.04 h, and 237.57 ± 3.41 h, respectively (Table 2). For *D. cornuta*, the shortest larval development time was 249.37 ± 4.88 h, 154.59 ± 1.81 h, 108.80 ± 2.65 h, and 86.04 ± 3.91 h, whereas the shortest intra-puparial development time was 593.37 ± 4.75 h, 414.23 ± 3.51 h, 261.02 ± 3.16 h, and 236.52 ± 2.66 h, respectively (Table 3). The above developmental time of both species were gradually shortened with increasing temperature.

Both species were able to grow and develop normally at different temperatures, with pupation rates > 87% and emergence rates > 81%.

### 3.2. Changes in Larval Body Length of Two Necrophagous Phorid Flies

**Figure 1:**
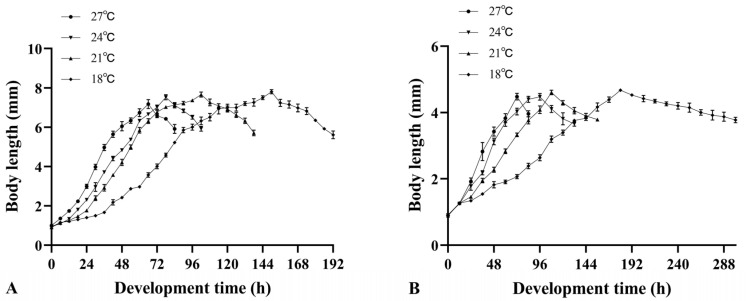
Larval body length changes in two phorid flies in sandy loam with 20% moisture content at various constant temperatures: (**A**) *Megaselia scalaris*, and (**B**) *Dohrniphora cornuta*.

The larval body lengths of both species increased rapidly during the feeding period and subsequently decreased after reaching the maximum values at 18, 21, 24, and 27 °C (Figure 1).

**Figure 2:**
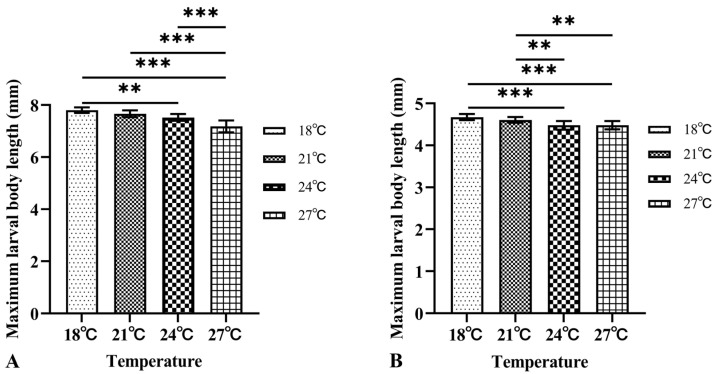
Maximum larval body length of two phorid flies in sandy loam with 20% moisture content at various constant temperatures: (**A**) *Megaselia scalaris*, and (**B**) *Dohrniphora cornuta*. ** represents *p* < 0.01, *** represents *p* < 0.001.

As the temperature increased, the time required for *M. scalaris* larvae to reach their maximum body length was 150 h, 102 h, 78 h, and 66 h, and the corresponding maximum body length was 7.80 ± 0.11 mm, 7.66 ± 0.13 mm, 7.52 ± 0.13 mm, and 7.18 ± 0.23 mm, respectively. The time taken by the larvae of *D. cornuta* to reach their maximum body length was 180 h, 108 h, 96 h, and 72 h, and the maximum body lengths were 4.67 ± 0.04 mm, 4.61 ± 0.07 mm, 4.48 ± 0.10 mm, and 4.48 ± 0.10 mm, respectively. There were significant differences in the maximum average larval body length of both species at different temperatures (Figure 2).

Regression equations were established for both species, with larval body length (mm) as the independent variable and development time after hatching (hours) as the dependent variable (Table 4 and Table 5). A cubic curve relationship was identified between these two variables in sandy loam with 20% moisture content at various constant temperatures.

### 3.3. Intra-Puparial Development of Two Necrophagous Phorid Flies

The pupae of *M. scalaris* and *D. cornuta* were collected and dissected at different developmental times across temperature conditions ranging from 18 °C to 27 °C. Based on the progressive changes in the external morphological characteristics, the intra-puparial period of both species was divided into 12 substages: (1) Protrusion of respiratory horns, (2) Segmentation of thorax and abdomen, (3) Differentiation of dorsal muscle of thorax and segment of abdomen, (4) Pupal cuticle detached from abdominal end, (5) Light yellow eye, (6) Scutellum, (7) Light yellow leg, (8) Light brown leg, (9) Brown leg, (10) Red-brown eye, (11) Black eye, and (12) Black wing. The above 12 substages apply to both sexes. These substages are described in detail below:

Stage 1: Protrusion of respiratory horns

**Figure 3:**
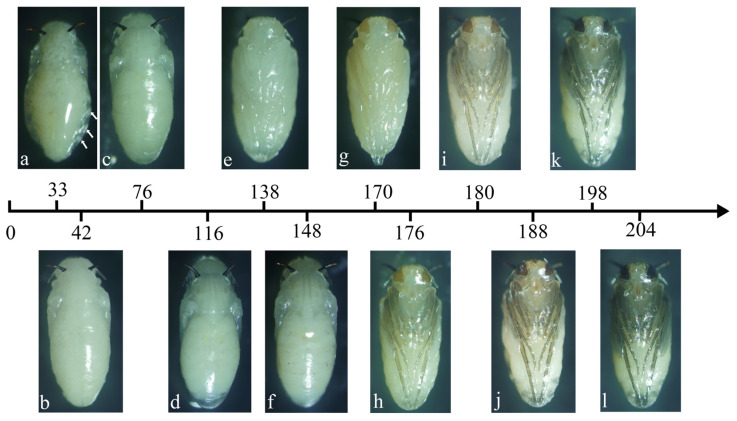
Developmental timeline of female *Megaselia scalaris* during the intra-puparial period at 27 °C and 20% moisture content. (**a**) Protrusion of respiratory horns stage, the arrows indicate the third larva remains; (**b**) Segmentation of thorax and abdomen stage; (**c**) Differentiation of dorsal muscle of thorax and segment of abdomen stage; (**d**) Pupal cuticle detached from abdominal end stage; (**e**) Light yellow eye stage; (**f**) Scutellum stage; (**g**) Light yellow leg stage; (**h**) Light brown leg stage; (**i**) Brown leg stage; (**j**) Red-brown eye stage; (**k**) Black eye stage; (**l**) Black wing stage.

**Figure 4:**
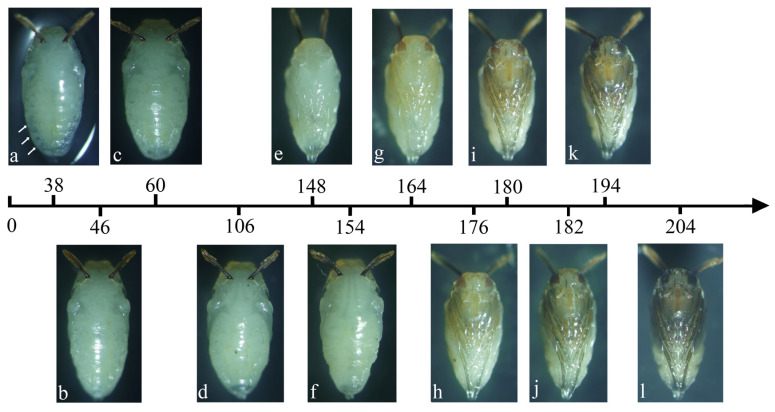
Developmental timeline of female *Dohrniphora cornuta* during the intra-puparial period at 27 °C and 20% moisture content. (**a**) Protrusion of respiratory horns stage, the arrows indicate the third larva remains; (**b**) Segmentation of thorax and abdomen stage; (**c**) Differentiation of dorsal muscle of thorax and segment of abdomen stage; (**d**) Pupal cuticle detached from abdominal end stage; (**e**) Light yellow eye stage; (**f**) Scutellum stage; (**g**) Light yellow leg stage; (**h**) Light brown leg stage; (**i**) Brown leg stage; (**j**) Red-brown eye stage; (**k**) Black eye stage; (**l**) Black wing stage.

The pupa has everted its respiratory horns. The remains of the third larva can be seen (Figure 3a and Figure 4a, arrows).

Stage 2: Segmentation of thorax and abdomen

At the dorsal view, the abdomen begins to develop and separate from the thorax (Figure 3b and Figure 4b).

Stage 3: Differentiation of dorsal muscle of thorax and segment of abdomen

The head, thorax, and abdomen of the pharate adult are obvious. The muscles of the thorax begin to differentiate. The dorsal segment of the abdomen begins to develop (Figure 3c and Figure 4c).

Stage 4: Pupal cuticle detached from abdominal end

The dorsal segments of the abdomen of the pharate adult differentiate, with its terminal segment clearly separated from the pupal cuticle (Figure 3d and Figure 4d).

Stage 5: Light yellow eye

At the ventral view, the eye cup bears a layer of bright yellow membrane at its perimeter (Figure 3e and Figure 4e).

Stage 6: Scutellum

At the dorsal view, the scutellum begins to differentiate (Figure 3f and Figure 4f).

Stage 7: Light yellow leg

At the ventral view, the color of the leg changes from colorless to light yellow (Figure 3g and Figure 4g).

Stage 8: Light brown leg

The leg color changes to light brown (Figure 3h and Figure 4h).

Stage 9: Brown leg

The compound eyes are brown and the leg color changes to brown (Figure 3i and Figure 4i).

Stage 10: Red-brown eye

The color of the compound eyes deepens and becomes reddish brown (Figure 3j and Figure 4j).

Stage 11: Black eye

The compound eyes are black and the folded wings are gray (Figure 3k and Figure 4k).

Stage 12: Black wing

The compound eyes are black and the folded wings turn black (Figure 3l and Figure 4l).

The time required for both species to develop to different substages decreased significantly with increasing temperature (Table 6 and Table 7).

## 4. Discussion

Soil type and moisture have been shown to significantly affect insect development [42,43,44,45,46,47], so the effect of soil type and moisture on insect development should be considered in addition to temperature when using developmental data from necrophagous insects to determine the time of death. Therefore, sandy loam was used in this study because it is a common soil type in the Shenyang area. The 20% moisture content was chosen because low humidity is more suitable for the growth and development of the phorid fly [44], as well as the moisture content of the soil (about 30 cm depth) collected from the Shenyang University campus and the poplar forest next to the Shenbei Expressway was determined to be about 20% in summer and autumn (our unpublished data). The mean daily soil temperature at 30 cm depth in Shenyang ranged from 1.8 °C to 23.3 °C between May and November [27]. Therefore, we set the experimental temperature range at 18–27 °C, which is suitable for phorid fly growth.

Insects are poikilotherms and their development is strongly influenced by temperature. The development time of both phorid flies in soil decreases with increasing temperature. Zuha and Omar [29] reported that the larval development time of *M. scalaris* feeding on cow liver in Kuala Lumpur, Malaysia, was 82.50 ± 0.00 h and 76.00 ± 0.00 h at 25 and 27 °C, respectively, and Zhang et al. [31] reported that the larval development time of *M. scalaris* in Suzhou, China, was approximately 107 h and 79.9 h at 25 °C and 28 °C, respectively. Inconsistent with their results, in the present study, the development time of *M. scalaris* was 90.28 ± 3.54 h and 63.04 ± 3.45 h at 24 °C and 27 °C, respectively. Wu et al. [35] reported that the development time of *D. cornuta* larvae was 186.81 ± 2.33 h, 169.15 ± 2.48 h, and 145.26 ± 2.31 h at 21 °C, 24 °C, and 27 °C (75% relative humidity), respectively. In our study, the corresponding development time of *D. cornuta* larvae was 154.59 ± 1.81 h, 108.80 ± 2.65 h, and 86.04 ± 3.91 h, which were significantly shorter than those reported by Wu et al. [35]. For *M. scalaris*, in this study, the intra-puparial development time was prolonged by 22.86 h and 28.55 h at 21 °C and 27 °C, respectively, compared with the data reported by Feng and Liu [30]. The intra-puparial development time of *D. cornuta* showed prolongations ranging from 21 h (at 21 °C) to 65.76 h (at 18 °C) relative to the findings of Feng et al. [4], although a reduction of 27.21 h was observed at 24 °C.

Larval body length is also an important day-age indicator. The larval body lengths of both species reared at 18–27 °C (with 20% soil moisture content) increased rapidly during the feeding period, then slightly decreased after reaching their maximum values. Greenberg and Wells [10] recorded the highest larval length of *M. scalaris* at about 6–7 mm at rearing temperatures (19–29 °C). Zuha and Omar [29] reported that the maximum mean larval length is 5.84 ± 0.56 mm (23 °C) and the lowest is 4.64 ± 0.77 mm (27 °C). Zhang et al. [31] observed a maximum larval length of about 7–7.5 mm at a temperature of 19–28 °C. However, in our study, the maximum body length of *M. scalaris* larvae ranged from 7.18 ± 0.23 mm to 7.80 ± 0.11 mm at a temperature of 18–27 °C. Wu et al. [35] reported that the average body length of *D. cornuta* larvae reached the maximum value at 21, 24, and 27 °C with 75% relative humidity for 144 h, 108 h, and 96 h, and the maximum body length was 4.91 ± 0.25 mm, 4.72 ± 0.08 mm, and 4.89 ± 0.12 mm, respectively. In the present study, the time required for *D. cornuta* larvae to reach the maximum body length at the same temperature was 108 h, 96 h, and 72 h, and the maximum body lengths were 4.61 ± 0.07 mm, 4.48 ± 0.10 mm, and 4.48 ± 0.10 mm, respectively. Therefore, it is essential to construct developmental data of phorid flies in simulated soil environments and not simply apply developmental data obtained under atmospheric temperature and humidity, which would lead to inaccurate inferences of PBI. However, food type also has an effect on fly development. Clark et al. [48] reported that larvae of *L. sericata* grew significantly faster when reared on pig tissue compared to cow tissue, and when reared on lung and heart compared to liver. Similarly, Bambaradeniya et al. [49] found the larval development duration of *L. sericata* was longest on heart tissue and shortest on skeletal muscle. El-Moaty and Kheirallah [50] investigated the effect of seven cow tissues (liver, brain, heart, lung, kidney, intestine, and minced meat) on the larval growth of *L. sericata*. They found significant differences in larval duration and body length between different tissues, with the smallest larvae obtained from those reared on heart tissue. In addition, the investigations on different geographical populations of *L. sericata* [51], *Chrysomya megacephala* [52,53], and *Cochliomyia macellaria* [54] indicate that the regional variation in developmental times exists within blow fly species. Therefore, the differences in development time and body length reported for these two phorid flies across the literature may also be due to food type and geographical population.

It is a more practical method to determine the intra-puparial age of necrophagous flies by dissecting the puparium and observing morphological changes in the pupa and pharate adult [4,55,56,57,58]. Feng and Liu [30] dissected *M. scalaris* pupae under six different constant temperature conditions (18–33 °C, 75% relative humidity) and subdivided the *M. scalaris* intra-puparial period into 10 substages (12 h intervals) based on the external morphological characteristics. Feng et al. [4] subdivided the *D. cornuta* intra-puparial period into nine substages (12–48 h intervals) under seven different constant temperature conditions (15–33 °C, 75% relative humidity). Compared to their results, due to the use of a shorter time interval (2 h or 4 h), we found that during the black eye stage, the compound eye was black first, and the wing then changed from gray to black. Thus, the black eye stage was followed by the black wing stage. Similarly, we separated the pupal cuticle detached from the abdominal end stage from the differentiation of the dorsal muscle of the thorax and the segment of the abdomen stage. The previous yellow eye stage [4,30] was subdivided into three stages: light yellow eye, scutellum, and light yellow leg. Therefore, these two species’ intra-puparial period was separated into 12 substages. In addition, the durations of some stages, such as protrusion of respiratory horns and pupal cuticle detached from the abdominal end, are still very long, and even longer at low temperatures, so other methods are needed to subdivide these stages, especially ones that can be quantified. Recently, the use of gene expression level changes to predict the age of *Calliphora vicina* (Robineau-Desvoidy, 1830) [59], *Lucilia illustris* Meigen, 1826 [57], and *Sarcophaga peregrina* (Robineau-Desvoidy, 1830) [60] during the intra-puparial period has proven to be effective. Shang et al. [61] reported that the variation tendencies of attenuated total reflectance-Fourier transform infrared (ATR-FTIR) spectroscopy and cuticular hydrocarbons (CHCs) of *S. peregrina* pupae were time-dependent, and the infrared spectroscopy and hydrocarbons may be optimal for age estimation of pupae of forensically important flies. So, these methods should be used to improve the precision of intra-puparial age estimation of these two phorid flies in the future.

The developmental data and images of intra-puparial development for these two phorid flies in sandy loam soil at 20% moisture under constant temperatures (18–27 °C) in this study will be helpful in future burial environment forensic investigations in the Shenyang region of China and are expected to contribute to similar research in other regions. However, this study also has several limitations. One limitation was the inability to determine the development time of the eggs. This is because phorid fly eggs are very small, not easy to find in sandy loam soil, and easy to be destroyed during soil disturbance, which precluded direct observation of egg hatching. Larval and intra-puparial development times for both species at temperatures below 18 °C were not available due to insufficient sampling. Additionally, although observations were made at 2 h or 4 h intervals, due to the limitations of the number of samples, it is not possible to determine the exact time of each distinctive feature during the intra-puparial period, allowing only approximate estimations. Soil type also significantly influences insect development [43,44,45], yet this study provides development data of both species only in one soil type. These limitations constrain the applicability of the current dataset. Therefore, future research should encompass investigations at lower temperatures (<18 °C) or fluctuating temperature regimes and under more soil types and moisture levels.

## 5. Conclusions

This study presents the first data on larval and intra-puparial development time, larval body length, and intra-puparial developmental changes in *M. scalaris* and *D. cornuta* in sandy loam with 20% moisture content at 18, 21, 24, and 27 °C. The development time of both species was gradually shortened with increasing temperature. There was a cubic curve relationship between larval body length and development time of the two species. According to the visible external morphological characteristics, the intra-puparial period of both species was divided into 12 developmental stages with detailed descriptions. The developmental data and pictures in this study will be helpful in estimating the PBI in future forensic investigations based on the juvenile age of *M. scalaris* and *D. cornuta*.

## Figures and Tables

**Table 1:** Summary of forensically important phorid flies.

Corpse Types	Species	Collection Environments	References
Human remains	*Conicera tibialis* Schmitz, 1925	indoors	[6]
		burial	[7]
	*Conicera similis* Haliday, 1833	burial	[8]
	*Dohrniphora cornuta* (Bigot, 1857)	indoors	[9]
		burial	[9]
	*Megaselia scalaris* (Loew, 1866)	indoors	[6,10,11,12,13]
		burial	[14,15,16]
	*Megaselia abdita* Schmitz, 1959	indoors	[6,10,17]
		burial	[18]
	*Megaselia rufipes* (Meigen, 1804)	indoors	[18]
	*Megaselia spiracularis* Schmitz, 1938	indoors	[13]
	*Megaselia curtineura* (Brues, 1909)	indoors	[13]
	*Triphleba opaca* (Meigen, 1830)	burial	[5]
	*Triphleba nudipalpis* (Schmitz, 1922)	burial	[19]
Animal remains	*Dahliphora sigmoides* Schmitz, 1923	waste bin	[20]
	*Diplonevra funebris* (Meigen, 1830)	outdoor	[21]
	*Diplonevra peregrina* (Wiedemann, 1830)	outdoor	[22,23]
	*Diplonevra florea* (Fabricius, 1794)	outdoor	[5]
	*Dohrniphora incisuralis* (Loew, 1866)	burial	[24]
	*Gymnoptera simplex* (Brues, 1905)	waste bin	[20]
	*Metopina subarcuata* Borgmeier, 1963	burial	[24]
	*Puliciphora borinquenensis* Wheeler, 1906	luggage and garbage bin	[25]
	*Puliciphora beckeri* Meijere, 1907	luggage and garbage bin	[25]
	*Puliciphora obtecta* Meijere, 1912	luggage and garbage bin	[25]
	*Spiniphora* sp.	waste bin	[20]
Animal muscle tissues	*Megaselia giraudii* (Egger, 1862)	outdoor	[26]
	*Metopina sagittata* Liu, 1995	burial	[27]

**Table 2:** The developmental time of *Megaselia scalaris* in sandy loam with 20% moisture content at various constant temperatures (mean ± SD).

Temperature (°C)	Feeding Period	Larval Period	Intra-Puparial Period	Pupation Rate (%)	Emergence Rate (%)
18	124.74 ± 0.88 d	165.18 ± 2.96 d	606.67 ± 3.38 d	87.67 ± 0.04 a	93.50 ± 0.04 b
21	86.37 ± 3.86 c	119.72 ± 2.74 c	404.62 ± 3.28 c	97.00 ± 0.03 b	96.00 ± 0.04 b
24	67.44 ± 3.50 b	90.28 ± 3.54 b	269.22 ± 6.04 b	90.00 ± 0.04 a	93.00 ± 0.05 b
27	45.47 ± 1.74 a	63.04 ± 3.45 a	237.57 ± 3.41 a	92.01 ± 0.04 a	81.50 ± 0.09 a

Different letters indicate significant differences (*p* < 0.05).

**Table 3:** The developmental time of *Dohrniphora cornuta* in sandy loam with 20% moisture content at various constant temperatures (mean ± SD).

Temperature (°C)	Feeding Period	Larval Period	Intra-Puparial Period	Pupation Rate (%)	Emergence Rate (%)
18	169.03 ± 3.39 d	249.37 ± 4.88 d	593.37 ± 4.75 d	89.68 ± 0.03 a	87.00 ± 0.04 a
21	116.77 ± 2.52 c	154.59 ± 1.81 c	414.23 ± 3.51 c	92.67 ± 0.05 a	95.00 ± 0.03 b
24	80.04 ± 3.01 b	108.80 ± 2.65 b	261.02 ± 3.16 b	92.00 ± 0.04 a	91.00 ± 0.05 ab
27	62.53 ± 5.80 a	86.04 ± 3.91 a	236.52 ± 2.66 a	90.00 ± 0.05 a	88.00 ± 0.08 a

Different letters indicate significant differences (*p* < 0.05).

**Table 4:** Equations of the relationship between the body length of *Megaselia scalaris* larvae in sandy loam with 20% moisture content and the time at various constant temperatures.

Temperature (°C)	Equation	R^2^	*F*	*p*
18	Y = −0.415X^3^ + 4.225X^2^ + 11.742X − 4.352	0.807	454.996	<0.001
21	Y = −0.616X^3^ + 7.255X^2^ − 7.836X + 10.108	0.769	262.054	<0.001
24	Y = −0.393X^3^ + 4.747X^2^ − 3.263X + 5.675	0.869	388.120	<0.001
27	Y = −0.127X^3^ + 1.772X^2^ + 3.969X − 2.196	0.844	264.044	<0.001

**Table 5:** Equations of the relationship between the body length of *Dohrniphora cornuta* larvae in sandy loam with 20% moisture content and the time at various constant temperatures.

Temperature (°C)	Equation	R^2^	*F*	*p*
18	Y = −15.262X^3^ + 122.161X^2^ − 224.632X + 134.277	0.770	285.720	<0.001
21	Y = −6.136X^3^ + 48.402X^2^ − 76.542X + 41.802	0.838	235.351	<0.001
24	Y = −5.562X^3^ + 42.510X^2^ − 65.585X + 33.472	0.754	118.426	<0.001
27	Y = −0.456X^3^ + 4.212X^2^ + 9.413X − 8.719	0.917	278.067	<0.001

**Table 6:** Time of distinctive features appearance of *Megaselia scalaris* during the intra-puparial period at different temperatures.

Temperature (°C)	Time of Distinctive Features Appearance (h)											
	a	b	c	d	e	f	g	h	i	j	k	l
18	82	126	154	204	324	350	412	432	450	464	490	514
21	54	90	138	180	228	252	286	306	316	324	332	340
24	40	64	96	116	166	176	192	202	208	212	228	232
27	33	42	76	116	138	148	170	176	180	188	198	204

**Table 7:** Time of distinctive features appearance of *Dohrniphora cornuta* during the intra-puparial period at different temperatures.

Temperature (°C)	Time of Distinctive Features Appearance (h)											
	a	b	c	d	e	f	g	h	i	j	k	l
18	84	120	176	240	330	382	402	452	466	480	494	512
21	59	90	114	168	230	262	278	312	320	328	336	344
24	43	78	98	120	180	192	210	220	224	230	234	248
27	38	46	60	106	148	154	164	176	180	182	194	204

## Data Availability

The original contributions presented in this study are included in the article. Further inquiries can be directed to the corresponding author.
